# Developmental Pathways Linking Maternal Depressive Symptoms with Parenting Quality and Preschool Children’s Screen Time: The Role of Children’s Behavioural Problems

**DOI:** 10.3390/children13091144

**Published:** 2026-08-26

**Authors:** Stefan Kurbatfinski, Rahul Gandhi, Deborah Dewey, Brenda M. Y. Leung, Nicole Letourneau

**Affiliations:** 1 Alberta Children’s Hospital Research Institute, Owerko Centre, Calgary, AB T2N 1N4, Canada; stefan.kurbatfinski@ucalgary.ca (S.K.); dmdewey@ucalgary.ca (D.D.); 2 Department of Community Health Sciences, Cumming School of Medicine, University of Calgary, Calgary, AB T2N 1N4, Canada; 3 Faculty of Sciences, Data Science and Analytics, University of Calgary, Calgary, AB T2N 1N4, Canada; 4 Hotchkiss Brain Institute, University of Calgary, Calgary, AB T2N 1N4, Canada; 5 Departments of Pediatrics and Community Health Sciences, Cumming School of Medicine, University of Calgary, Calgary, AB T2N 1N4, Canada; 6 Faculty of Health Sciences, University of Lethbridge, Lethbridge, AB T1K 3M4, Canada; 7 Faculty of Nursing, University of Calgary, Calgary, AB T2N 1N4, Canada; 8 Departments of Pediatrics, Psychiatry, and Community Health Sciences, Cumming School of Medicine, University of Calgary, Calgary, AB T2N 1N4, Canada

**Keywords:** maternal depressive symptoms, children’s behavioural problems, parenting quality, children’s screen time, the APrON Study, longitudinal

## Abstract

**Highlights:**

**What are the main findings?**

- In a low-risk sample, maternal mean perinatal depressive symptoms were directly associated with nurturant parenting quality, while externalizing symptoms mediated the association with hostile parenting quality.
- In an extended model, externalizing symptoms mediated the association between maternal depressive symptoms and child screen time during the pre-bedtime period.

**What is the implication of the main findings?**

- Primary prevention of maternal depressive symptoms, even at subclinical levels, may be important for promoting nurturant and reducing hostile parenting.
- Among children of mothers experiencing depressive symptoms, targeting externalizing problems may help reduce later hostile parenting and child screen time.

**Abstract:**

Background: Maternal depressive symptoms have been associated with adverse child and family outcomes, including child behavioural problems, suboptimal parenting quality, and increased child screen time. However, the developmental pathways linking these factors remain poorly understood. The first model of this study examined whether children’s behavioural problems mediated associations between maternal depressive symptoms and later parenting quality (i.e., nurturant, hostile, consistent). The second model then investigated whether these pathways extended to preschool children’s screen time during the pre-bedtime period. Methods: Longitudinal data from the APrON Study (N = 373 dyads) were analyzed. A mean score for maternal depressive symptoms (EPDS) was derived using data collected across pregnancy and 3, 6, and 12 months postpartum. At age 3 years, child behaviour was assessed using the Child Behavior Checklist. At 5 years, parenting quality was measured using the Parenting Scale, and children’s screen time (frequency of use before bedtime) was assessed using a single maternal-reported item developed by the APrON team. Parallel and parallel serial mediation models were estimated using structural equation modeling. Findings: Maternal depressive symptoms were indirectly associated with more hostile parenting through children’s externalizing problems (*p* < 0.01, 95% CI [0.02, 0.11]) and directly associated with less nurturant parenting (*p* = 0.02, 95% CI [−0.19, −0.02]). Results from the parallel serial mediation model indicated that maternal depressive symptoms were indirectly associated with greater child screen time via children’s externalizing problems (*p* = 0.02, 95% CI [0.01, 0.06]). Significance: These findings suggest that children’s externalizing problems may represent a plausible pathway linking maternal depressive symptoms with later maternal hostile parenting and preschool children’s screen time.

## 1. Introduction

Perinatal depression in mothers, defined as depression experienced during pregnancy and up to 1 year postpartum, consistently emerges as a robust predictor of adverse child and maternal health outcomes [1,2]. Maternal perinatal depressive symptoms have been strongly associated with children’s behavioural problems, especially during the preschool (two to five years old) period [3], which may subsequently contribute to downstream family and child outcomes [4]. Some downstream outcomes of interest include parenting quality [5,6,7,8,9] and child screen time [10,11,12]. However, the developmental pathways through which maternal perinatal depressive symptoms influence later parenting quality and children’s screen time via children’s behavioural problems remain unexplored, highlighting the need for longitudinal research examining these interconnected developmental processes. 

These developmental pathways are important because parenting quality has been associated with a range of family and child health outcomes across the lifespan, including children’s mental and physical health [13], cognition development [13], academic achievement [14], and parental mental health [15]. Suboptimal parenting quality has also been associated with increased child screen time within the home environment [16]. Conversely, excessive screen time may negatively influence children’s sleep quality, contribute to overstimulation, and function as an addictive outlet that interferes with the development of self-regulation and displaces developmentally important activities [17,18]. Screen time around the bedtime period may be particularly important to investigate as parents may experience challenges in regulating children in preparation for bedtime [19,20]. Therefore, screen time may be used to help with the transition to bedtime [21,22,23] despite evidence that it may disrupt sleep quality [24,25]. Screen time during this period may also displace developmentally supportive activities, such as shared book reading [26]. These patterns may be more prominent among mothers experiencing depressive symptoms [27] or children displaying behavioural problems [28], making the pre-bedtime period an important context in which to examine children’s screen time. 

The preschool period represents a particularly sensitive developmental stage during which behavioural regulation, parenting practices, and screen time habits may become increasingly established, making it an important period for examining these interconnected developmental pathways [29,30]. Accordingly, this study examined whether preschool children’s behavioural problems mediated associations between maternal depressive symptoms and subsequent parenting quality and whether these developmental pathways extended to preschool children’s screen time.

### 1.1. Maternal Depression and Children’s Behavioural Problems

Children’s behavioural problems include internalizing and externalizing symptoms. Internalizing problems are inwardly expressed, reflecting behaviours such as anxiety and depression [31]. Generally reflecting psychological distress, internalizing symptoms are considered less observable [31]. Conversely, externalizing problems are outwardly expressed, including behaviours such as screaming and hitting [32]. As they are more physical in nature, externalizing symptoms have generally been described as easier to detect [32]. Despite manifesting differently, both types of behavioural problems are linked to various adverse outcomes, including health sequelae, engagement in risky health behaviours (e.g., substance use), and lower socioeconomic attainment [23,31,32]. Of relevance, both types may be influenced by maternal depression prenatally and postnatally [3,30,33]. 

Fetal programming mechanisms support that maternal depressive symptoms during pregnancy are associated with alterations in offspring brain development, which, in turn, have been linked to behavioural problems in early childhood [30,33]. Postnatally, cross-sectional and longitudinal studies have demonstrated positive associations between maternal depression and children’s internalizing and externalizing problems, particularly during the preschool period (two to five years old) [3]. Children’s behavioural problems may therefore represent an important developmental consequence of maternal depressive symptoms that contributes to subsequent child and family outcomes; however, research has predominantly examined behavioural symptoms as outcomes rather than a potential developmental mechanism.

### 1.2. Child Behaviour as a Developmental Pathway Linking Maternal Depression to Parenting

Symptoms of maternal depression have been associated with reduced sensitivity to children in the absence of intervention, potentially resulting in suboptimal or neglectful responses [34,35,36,37]. Empirical evidence further suggests that depressive symptoms may disrupt physiological synchrony and mutually positive mother–child interactions [38]. Accordingly, maternal depressive symptoms have been identified as an important predictor of parenting quality [39,40,41], which can be described as nurturant, hostile, or consistent [42] (Table 1). Research has demonstrated that maternal depressive symptoms are associated with more hostile and inconsistent parenting quality [40,41], and to a lesser degree, with mothers’ nurturant parenting quality [39]. However, the extent to which children’s behavioural symptoms mediate these associations remains understudied. This gap is partly explained by the longstanding emphasis on parenting quality as a determinant of children’s behavioural problems rather than considering children’s behavioural problems as contributing to subsequent parenting quality [5,6].

Growing theoretical and empirical evidence supports the reciprocal nature of mother–child relationships, whereby child behaviour is believed to actively contribute to and shape parenting quality itself [5,6,45]. Children with greater behavioural difficulties may require more frequent behavioural management, elicit higher levels of parental stress, and reduce opportunities for positive parent–child interactions [4,46]. Consequently, parents may become less consistent, less nurturant, or more hostile in response to persistent behavioural challenges, in line with transactional models of parent–child development [4]. Empirical evidence provides support for this theory, indicating that more reactive or disruptive child behaviour profiles, typically characterized by externalizing problems, have been observed to elicit harsher and/or less involved parenting behaviours [7,8,9], although these associations have not been consistently observed [47]. Conversely, findings regarding internalizing problems are less consistent, with associations generally only observed for nurturant parenting quality [9] and characterized as weak in magnitude [48]. 

These differences suggest that internalizing and externalizing problems may represent distinct developmental pathways through which maternal depressive symptoms influence later parenting quality. Given that maternal depressive symptoms, particularly during the perinatal period, are associated with preschool children’s behavioural problems [3], and that children’s behavioural problems may subsequently influence parenting quality through reciprocal parent–child interactions [5,6], children’s behavioural problems may represent an important yet understudied mediator of the association between maternal depressive symptoms and parenting quality.

### 1.3. Child Behaviour as a Developmental Pathway Linking Maternal Depression to Child Screen Time

Given that screen time can negatively affect children’s health and development, identifying early family and child factors that contribute to screen time represents an important area of investigation [17,18]. Maternal depressive symptoms [49], children’s behavioural problems [50], and suboptimal parenting quality [51] have each been associated with children’s emotional dysregulation, which may increase the likelihood that children seek comfort or regulation through alternative sources [52]. One such source may be screen time, which has been identified as a potential strategy for emotional regulation in young children [10], especially during the transition to bedtime [21,22,23].

Children’s screen time may increase following the emergence of behavioural problems [10], either as a means of supporting self-regulation or because parents may use screen-based activities to manage children’s behavioural difficulties [12,53]. Mothers experiencing depressive symptoms may also engage in more screen time themselves [54], which is a robust, strong predictor of their children’s screen time [55]. Similarly, preschoolers experiencing less optimal parenting quality may turn to screens as a means of self-regulation [11,12], suggesting a more complex, developmental pathway linking parenting quality and child screen time. Accordingly, maternal depressive symptoms may contribute to increased preschool screen time both directly and indirectly through their associations with children’s behavioural problems and parenting quality [12,53]. Despite the potential of these independent and serial mediation pathways to elucidate important developmental mechanisms and identify potentially modifiable targets for reducing excessive screen time, they remain unexplored to our knowledge.

### 1.4. Purpose of the Study

**Figure 1:**
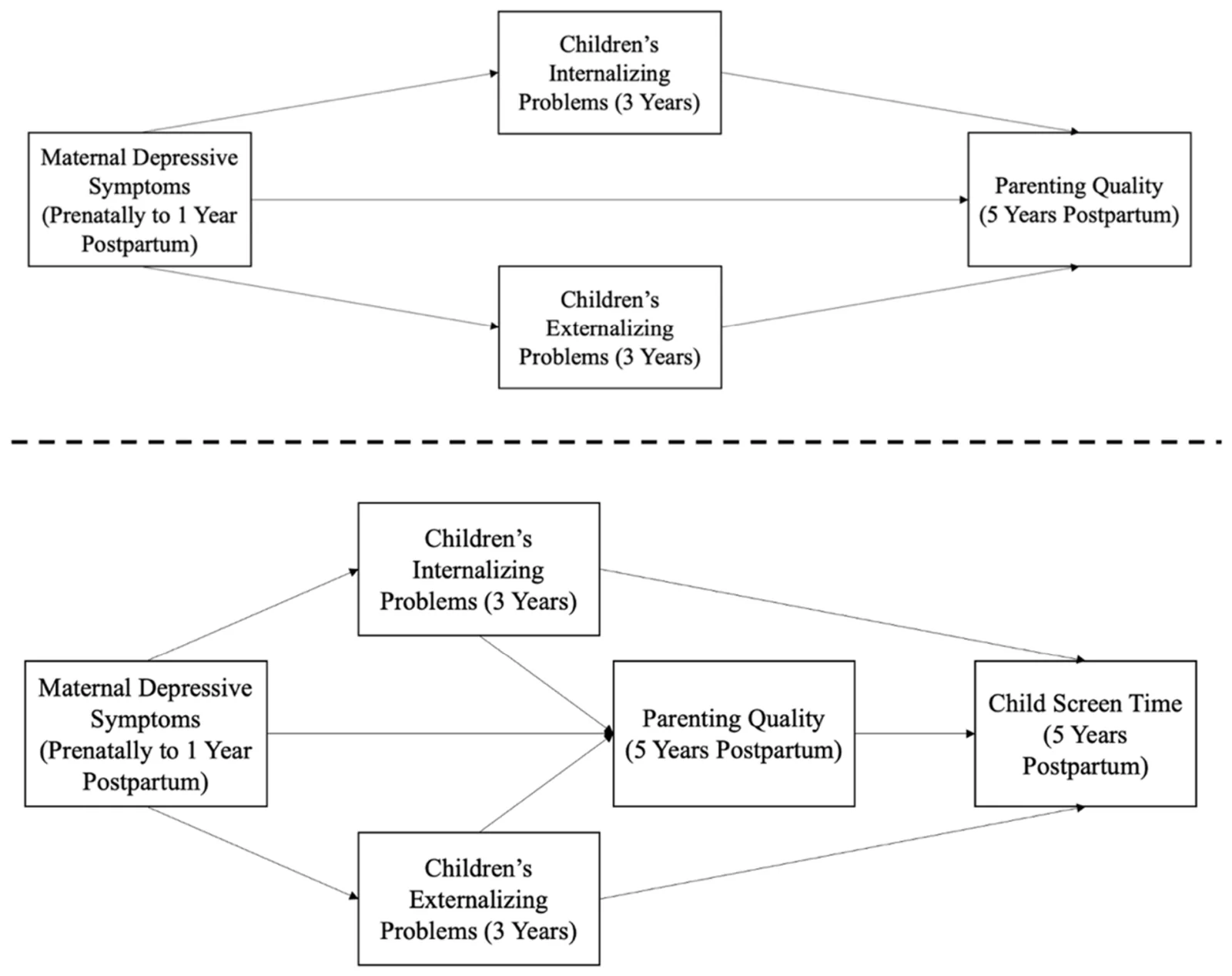
Proposed mediation models: top panel = mediation model 1 (parallel mediation) and bottom panel = mediation model 2 (parallel serial mediation). Total effects, total indirect effects, and direct effects were also estimated across models.

The purpose of this study was two-fold. First, we examined whether maternal depressive symptoms assessed across the perinatal period were associated with parenting quality at 5 years postpartum, and whether the association is mediated through children’s behavioural problems at 3 years of age (Figure 1, Top Panel). Specifically, the first model (parallel mediation) addressed the following research questions: (1) “What is the association between mothers’ depressive symptoms and their parenting quality at 5 years postpartum?” and (2) “Do children’s behavioural problems mediate the association between maternal depressive symptoms and children’s behavioural problems?” We hypothesized that greater maternal depressive symptoms would be associated with more hostile, less consistent, and less nurturant parenting, and that these associations would be partially explained through greater child internalizing and externalizing problems.

Second, we examined whether the first developmental model extended to child screen time, by testing independent and serial mediation pathways linking maternal depressive symptoms with child screen time through children’s behavioural problems and parenting quality (Figure 1, Bottom Panel). Specifically, the second model (parallel serial mediation) addressed the following research questions: (1) “What is the association between mothers’ depressive symptoms and child screen time at 5 years postpartum?” and (2) “Do children’s behavioural problems and parenting quality independently and serially mediate the association between maternal depressive symptoms and child screen time?” We hypothesized that greater maternal depressive symptoms would be associated with increased child screen time through both independent indirect effects of children’s behavioural problems and parenting quality, as well as serial indirect effects whereby greater maternal depressive symptoms would predict more behavioural problems in children, which would subsequently contribute to a less optimal parenting quality and, ultimately, increased child screen time.

## 2. Methods

### 2.1. Study Design

This study used data from the APrON Study, an ongoing longitudinal and prospective cohort taking place in two metropolitan cities in the province of Alberta, Canada [56]. Data on maternal depression was collected during each trimester of pregnancy, followed by assessments at 3, 6, and 12 months postpartum. Children’s behavioural problems were measured at the three-year follow-up, whereas parenting quality and child screen time were measured at the 5-year follow-up. Although the dataset is not currently housed in an open-access repository, data may be obtained upon reasonable request from the corresponding author (NL). Further details about the APrON Study are accessible elsewhere [56].

Ethical approval for this study was obtained from two institutional review boards: the University of Calgary’s Conjoint Health Research Ethics Board (REB14-1702) and the University of Alberta’s Health Research Ethics Biomedical Panel (Pro00002954). Written informed consent from participants was obtained at enrolment, with ongoing consent reaffirmed at each subsequent time point throughout the five-year follow-up period to ensure continued ethical oversight.

### 2.2. Sample and Inclusion Criteria

To be recruited in the APrON Study, pregnant women had to be less than 27 weeks’ gestation, live in or near one of the two study cities, and be able to attend scheduled visits at either the University of Calgary or the University of Alberta. Also, pregnant women had to be at least 16 years of age, biologically related to the child, fluent in English (reading and speaking), and plan to remain in the area for up to three months postpartum. Women who did not read or write in English or did not plan to remain in the study area until 3 months postpartum were excluded. Among the 2189 participants who enrolled in the APrON Study in either the first or second trimester of pregnancy, 1353 responded to the behavioural problems questionnaire provided at the 3-year follow-up (response rate 61.81%). Of the 1353 participants who provided data on their children’s behavioural problems, only 373 had data available for child screen time and nurturant, hostile, and consistent parenting quality at 5 years and were included in this study (See Supplementary Materials Figure S1 for a participant flow diagram).

### 2.3. Included Variables and Measures

#### 2.3.1. Predictor 

Maternal depressive symptoms were assessed longitudinally using the Edinburgh Postnatal Depression Scale (EPDS), a widely validated 10-item self-report questionnaire designed to screen for prenatal and postpartum depression [57]. Scores above 12 suggest that Canadian mothers are at clinical risk of depression [58]. As part of routine data collection, mothers completed the EPDS at multiple timepoints (each trimester of pregnancy and at 3, 6, and 12 months postpartum). EPDS scores were averaged across the time points to capitalize on repeated measures data, producing a single, mean estimate. The EPDS is a well-established measure in perinatal research, exhibiting strong psychometric validity for detecting maternal depressive symptoms [57,59,60]; therefore, it is considered a reliable tool of assessing maternal depressive symptoms.

#### 2.3.2. First Model Outcome (And Subsequent Mediator)

Maternal parenting quality (nurturant, hostile, and consistent) was assessed using items from the Parenting Scale, which was derived from the National Longitudinal Survey of Children and Youth [42]. The Parenting Scale is a parent self-report measure of parenting quality. It is composed of 26 items and uses a 5-point Likert scale (0 = never to 4 = always). Three parenting quality subscales were derived for this study: positive parenting (proxy for nurturant; five items), hostile parenting (seven items, 1 of which is reverse-scored), and consistent parenting (five items, 3 of which are reverse-scored; [42]). Subscale scores are calculated as the sum of relevant items. Possible scores for nurturant and consistent parenting quality range from 0 to 20, and for hostile parenting quality from 0 to 28; higher scores indicate greater levels of each respective parenting quality [42]. Internal consistency reliability for each subscale was evaluated using McDonald’s omega, which indicated moderate internal consistency for nurturant (ω = 0.74), hostile (ω = 0.78), and consistent (ω = 0.70) parenting quality.

#### 2.3.3. Second Model Outcome

Child screen time, focused on the pre-bedtime period, was assessed using a single item on a maternal-report questionnaire. Mothers were asked to report the number of days per week their child engaged in screen-based activities (e.g., television, smartphone, computer, tablet use) during the hour before going to bed. Responses ranged from 0 (no screen time before bedtime) to 7 (screen time before bed every day of the week). This single-item question was developed by the APrON team to briefly assess the frequency of children’s pre-bedtime screen time. 

#### 2.3.4. Mediators

Children’s internalizing and externalizing problems were assessed at approximately 3 years of age using the Child Behavior Checklist (CBCL) [61]. The CBCL evaluates multiple domains of child behavioural functioning during early childhood [61], and was developed using normative data from diverse populations, including the general population and clinical groups (i.e., children with neurodevelopmental disorders) within the United States [61]. Parents completed a questionnaire assessing a range of behaviours exhibited by their child. Raw scores for the Internalizing and Externalizing Composite scales were converted to age- and sex-standardized T-scores (M = 50, SD = 10), based on the normative sample [61]. T-scores above 64 indicate clinically elevated behavioural problems, while scores ranging from 60 to 63 indicated borderline or at-risk levels, and scores below 60 suggest minimal-to-no behavioural concerns [61]. Previous evaluations of the CBCL have consistently demonstrated strong reliability and construct validity [61].

#### 2.3.5. Covariates

Theory-driven covariates were added, including maternal self-identified ethnicity, highest level of education achieved, total household annual income, maternal age, child age at 5 years, gestational age and birthweight, and child sex assigned at birth [62,63,64,65,66,67]. Maternal self-identified ethnicity, highest level of education achieved, and total household annual income were collected at study enrolment. Maternal self-identified ethnicity was collapsed into a dichotomous variable, non-racialized (i.e., white) versus racialized (i.e., Chinese, Latin American, Black, or other), due to low variation. Gestational age, birthweight, and child sex assigned at birth were recorded at delivery using maternal reports and birth records; in cases of discrepancies, birth records were used. Maternal age and child age were derived by subtracting their dates of birth, respectively, from the date the 5-year assessment was completed. 

### 2.4. Missing Data

After merging the data for all relevant variables, the proportion of missing data was less than 10% for all variables except for hostile parenting quality (42.90% missingness). Patterns of missingness were examined using Little’s test, which examines whether data are missing completely at random or not [68]. Significance indicates that data were not missing completely at random [68], as was the case in this study (*p* = 0.03). Therefore, the data were assumed to be missing at random, and missing data were imputed using multiple imputation by chained equations, with 20 imputed datasets generated over 50 iterations, using predictive mean matching for continuous variables, proportional odds logistic regression for ordinal variables, polytomous logistic regression for nominal variables, and binary logistic regression for dichotomous variables [69]. Multiple imputation by chained equations is a suitable approach for addressing data of variables missing less than 50% of observations and that are deemed at least missing at random [69].

### 2.5. Data Analysis

Analyses were conducted using R version 4.5.2 [70]. Descriptive statistics were used to summarize sample demographics, including means and standard deviations for continuous variables and proportions for categorical variables. Summary statistics were computed for the main predictor, outcomes, mediators, and covariates to characterize the distributions and surface patterns in the data. 

All mediation analyses were conducted using structural equation modelling (SEM) in R with the *lavaan* [71] and *lavaan.mi* [72] packages. Model estimates and standard errors were pooled across the 20 imputed datasets using Rubin’s rules. For each model, all regression paths were estimated simultaneously, allowing direct, indirect, and total effects to be estimated within a single structural equation model. All relevant covariates were included in each regression equation, except child age at 5 years, which was included only in equations predicting 5-year outcomes (i.e., parenting quality and child screen time). Indirect effects were computed as the product of the constituent path coefficients. An alpha level of 0.05 was used for significance testing, and 95% confidence intervals were used to interpret statistical significance.

#### 2.5.1. First Model

To examine the first model, a parallel mediation model was estimated. Mothers’ mean perinatal depressive symptoms were entered as the predictor, children’s internalizing and externalizing problems were entered as parallel mediators, and parenting quality was entered as the outcome. Given that three different dimensions of parenting quality were investigated (i.e., hostile, nurturant, or consistent), three separate models were estimated, with each model including one parenting outcome. Indirect effects through children’s internalizing and externalizing problems were estimated simultaneously, with each indirect effect accounting for the covariance between the two mediators.

#### 2.5.2. Second Model

To examine whether the pathways identified in the first model extended to children’s screen time before bedtime, a parallel serial mediation model was estimated. Mothers’ mean perinatal depressive symptoms were entered as the predictor, children’s internalizing and externalizing problems were specified as parallel first-stage mediators, parenting quality was specified as an independent and second-stage serial mediator, and children’s screen time specified as the outcome. This model simultaneously estimated five indirect effects: (1) through internalizing problems alone, (2) through externalizing problems alone, (3) through parenting quality alone, (4) through internalizing problems followed by parenting quality, and (5) through externalizing problems followed by parenting quality. Internalizing and externalizing problems were allowed to covary, and direct paths from maternal depressive symptoms to parenting quality and children’s screen time, as well as from children’s behavioural problems to screen time, were estimated. Once again, separate models were estimated for nurturant, hostile, and consistent parenting.

## 3. Results

Demographic information for the sample (N = 373) is provided (Table 2). Most mothers self-identified as non-racialized (91%), with fewer self-identifying with a racialized ethnicity (~9). More than three-quarters (77%) of mothers reported having completed at least an undergraduate university degree. Just under two-thirds of households reported annual incomes exceeding $100,000 CAD. Distribution of child sex assigned at birth was relatively even, with 50.7% and 49.3% of children male and female, respectively.

### 3.1. Descriptive Statistics 

Descriptive statistics are provided (Table 3). As expected in a low-risk sample, the distribution of women’s maximum depressive symptoms was right-skewed, with most participants reporting low levels of depressive symptoms; scores ranged from 0 to 16.3, with a median of 4. Women generally reported high nurturant (mean = 14.97), low hostile (mean = 9.40), and low consistent (mean = 16.27) parenting quality. Children’s weekly screen time showed substantial variability with a bimodal distribution: the largest proportion (21%) of children were reported to engage in screen-based activities before bedtime seven days per week, followed closely by those with zero (19%) or one (19%) day of screen time (Table 3). Regarding child behavioural outcomes, the mean internalizing behavioural T score was 45.43 (SD = 8.76), and the mean externalizing behavioural T score was 45.48 (SD = 8.70). 

### 3.2. First Model: Parenting as the Outcome (Parallel Mediation)

A comprehensive statistical summary for the first models is provided (See Supplementary Materials Table S1). 

#### 3.2.1. Nurturant Parenting Quality

Results indicated that the indirect effects of maternal depressive symptoms on nurturant parenting quality through children’s externalizing (effect = −0.01, *p* = 0.33, 95% CI [−0.03, 0.01]) and internalizing (effect = 0.00, *p* = 0.98, 95% CI [−0.03, 0.03]) problems were not statistically significant. Similarly, the total indirect effect was not significant (effect = −0.01, *p* = 0.38, 95% CI [−0.03, 0.01]). However, maternal depressive symptoms demonstrated a significant direct effect on nurturant parenting quality (effect = −0.10, *p* = 0.02, 95% CI [−0.19, −0.02]). The total effect of maternal depressive symptoms was also significant (effect = −0.11, *p* < 0.01, 95% CI [−0.20, −0.03]), indicating an overall negative association.

#### 3.2.2. Hostile Parenting Quality

Results indicated that the indirect effect of maternal depressive symptoms on hostile parenting quality via children’s externalizing problems was statistically significant (effect = 0.07, *p* < 0.01, 95% CI [0.02, 0.11]). In contrast, the indirect effect via internalizing problems was not significant (effect = −0.02, *p* = 0.39, bootstrap 95% CI [−0.06, 0.02]). Contrast analysis indicated that these indirect effects differed significantly from one another (effect = −0.08, *p* = 0.02, 95% CI [−0.15, −0.01]). The total indirect effect was also statistically significant (effect = 0.05, *p* = 0.03, 95% CI [0.05, 0.09]). Neither the direct effect (effect = 0.04, *p* = 0.51, 95% CI [−0.08, 0.17], nor the total effect (effect = 0.09, *p* = 0.16, 95% CI [−0.03, 0.21]) of maternal depressive symptoms on hostile parenting quality was statistically significant. 

#### 3.2.3. Consistent Parenting Quality

Results indicated that the indirect effects of maternal depressive symptoms on consistent parenting quality through children’s externalizing (effect = −0.01, *p* = 0.23, 95% CI [−0.03, 0.01]) and internalizing (effect = 0.01, *p* = 0.42, 95% CI [−0.02, 0.04]) problems were not statistically significant. Similarly, the total indirect effect was not statistically significant (effect = −0.01, *p* = 0.91, 95% CI [−0.03, 0.02]). Neither the direct effect (effect = −0.07, *p* = 0.13, 95% CI [−0.16, 0.02]), nor the total effect (effect = −0.07, *p* = 0.11, 95% CI [−0.16, 0.02]) of maternal depressive symptoms on consistent parenting quality was statistically significant.

### 3.3. Second Model: Child Screen Time as the Outcome (Parallel Serial Mediation)

A comprehensive statistical summary for the second models is provided (See Supplementary Materials Table S2). 

#### 3.3.1. Serial Mediation via Child Behaviour Followed by Nurturant Parenting Quality

Serial indirect effects through internalizing behaviours (effect = 0.00, *p* = 0.96, 95% CI [−0.01, 0.01]) or externalizing problems (effect = 0.01, *p* = 0.39, 95% CI [−0.01, 0.01]), followed by nurturant parenting quality were not statistically significant. Similarly, the indirect effect through nurturant parenting quality alone was not significant (effect = 0.01, *p* = 0.24, 95% CI [−0.01, 0.02]). The indirect effect of maternal depressive symptoms on child screen time through children’s internalizing problems was also not significant (effect = −0.01, *p* = 0.35, 95% CI [−0.04, 0.01]), and the total indirect effect through internalizing problems was not significant (effect = −0.01, *p* = 0.35, 95% CI [−0.04, 0.01]). 

In contrast, an indirect effect via externalizing problems was statistically significant (effect = 0.03, *p* = 0.02, 95% CI [0.01, 0.06]), resulting in a significant total indirect effect through externalizing problems (effect = 0.03, *p* = 0.01, 95% CI [0.01, 0.06]). Contrast analysis indicated that the indirect effect through externalizing problems was larger than the indirect effect through internalizing problems; however, this difference did not reach statistical significance (effect = −0.05, *p* = 0.054, 95% CI [−0.09, 0.01]). A similar pattern was observed when comparing the total indirect effects through externalizing and internalizing problems (effect = −0.05, *p* = 0.052, 95% CI [−0.10, 0.01]). 

Finally, the total effect (effect = 0.08, *p* = 0.06, 95% CI [−0.01, 0.17]) and overall total indirect effect (effect = 0.03, *p* = 0.07, 95% CI [−0.01, 0.06]) approached statistical significance, whereas the direct effect of maternal depressive symptoms on child screen time was not significant (effect = 0.05, *p* = 0.22, 95% CI [−0.03, 0.14]).

#### 3.3.2. Serial Mediation via Child Behaviour Followed by Hostile Parenting Quality

Serial indirect effects though internalizing problems (effect = −0.01, *p* = 0.42, 95% CI [−0.01, 0.01]) or externalizing problems (effect = 0.01, *p* = 0.30, 95% CI [−0.01, 0.01]), followed by hostile parenting quality were not statistically significant. Similarly, the indirect effect through hostile parenting quality alone was not significant (effect = 0.01, *p* = 0.67, 95% CI [−0.01, 0.01]). The indirect effect of maternal depressive behaviours on child screen time through children’s internalizing problems was also not statistically significant (effect = −0.01, *p* = 0.41, 95% CI [−0.04, 0.02]), and the total indirect effect through internalizing problems was not significant (effect = −0.01, *p* = 0.39, 95% CI [−0.04, 0.02]). 

In contrast, an indirect effect through externalizing problems was statistically significant (effect = 0.03, *p* = 0.03, 95% CI [0.01, 0.06]), resulting in a significant total indirect effect through externalizing problems (effect = 0.03, *p* = 0.02, 95% CI [0.01, 0.06]). Contrast analysis suggested that the indirect effect through externalizing problems was larger than the indirect effect through internalizing problems; however, this difference did not reach statistical significance (effect = −0.04, *p* = 0.09, 95% CI [−0.10, 0.01]). A similar pattern was observed when comparing the total indirect effects through externalizing and internalizing problems (effect = −0.05, *p* = 0.07, 95% CI [−0.10, 0.01]). 

Finally, the total effect approached statistical significance (effect = 0.08, *p* = 0.08, 95% CI [−0.01, 0.17]), whereas the overall total indirect effect was not significant (effect = 0.02, *p* = 0.15, 95% CI [−0.01, 0.05]). The direct effect of maternal depressive symptoms on child screen time was also not significant (effect = 0.06, *p* = 0.21, 95% CI [−0.03, 0.15]).

#### 3.3.3. Serial Mediation via Child Behaviour Followed by Consistent Parenting Quality

Serial indirect effects through internalizing problems (effect = −0.01, *p* = 0.50, 95% CI [−0.01, 0.01]) or externalizing problems (effect = 0.01, *p* = 0.35, 95% CI [−0.01, 0.01]), followed by consistent parenting were not statistically significant. Similarly, the indirect effect though consistent parenting alone was not significant (effect = 0.01, *p* = 0.23, 95% CI [−0.01, 0.02]). The indirect effect of maternal depressive symptoms on child screen time through children’s internalizing problems was also not significant (effect = −0.01, *p* = 0.40, 95% CI [−0.04, 0.02]), and the total indirect effect through internalizing problems was not significant (effect = −0.01, *p* = 0.36, 95% CI [−0.04, 0.02]).

In contrast, the indirect effect through children’s externalizing problems was statistically significant (effect = 0.03, *p* = 0.02, 95% CI [0.01, 0.06]), resulting in a significant total indirect effect through externalizing problems (effect = 0.03, *p* = 0.02, 95% CI [0.01, 0.06]). Contrast analysis indicated that the indirect effect through externalizing problems was larger than that through internalizing problems; however, this difference was not statistically significant (effect = −0.04, *p* = 0.07, 95% CI [−0.09, 0.01]). A similar pattern was observed when comparing the total indirect effects through externalizing and internalizing problems (effect = −0.05, *p* = 0.055, 95% CI [−0.10, 0.01]). 

Finally, the total effect (effect = 0.08, *p* = 0.06, 95% CI [−0.01, 0.17]) and overall total indirect effect (effect = 0.03, *p* = 0.06, 95% CI [−0.01, 0.06]) approached statistical significance, whereas the direct effect of maternal depressive symptoms on child screen time was not significant (effect = 0.05, *p* = 0.24, 95% CI [−0.03, 0.14]).

## 4. Discussion

This longitudinal Canadian study examined specific developmental pathways linking perinatal maternal depressive symptoms (assessed across pregnancy and up to 1 year postpartum), children’s behavioural problems (at 3 years of age), parenting quality (at 5 years of age), and child screen time (operationalized as before bedtime at 5 years of age) through theoretically informed and plausible mediation pathways in a low-risk sample. The first model identified children’s externalizing problems as a potential psychosocial mechanism underlying the association between maternal depressive symptoms and more hostile parenting, supporting the reciprocal nature of mother–child relationships in which children actively contribute to and shape parenting quality [5,6,45]. The absence of significant direct and total effects further suggests that the association between maternal depressive symptoms and hostile parenting was primarily accounted for by this indirect pathway. 

In contrast, children’s behavioural problems did not appear to mediate the association between maternal depressive symptoms and nurturant parenting. However, significant direct and total effects indicated that maternal depressive symptoms were associated with less nurturant parenting in this sample. No significant associations or mediation pathways were identified for consistent parenting quality. 

Although our second model extended the pathways examined in the first model to investigate associations with child screen time at 5 years of age, no serial mediation pathways were identified. However, children’s externalizing problems consistently mediated the association between maternal depressive symptoms and child screen time across models, with this indirect effect appearing larger than that of internalizing problems. Considering the significant total effect and significant total indirect effect through externalizing problems, and non-significant direct effect observed across models, these findings suggest that the association between maternal depressive symptoms and child screen time was primarily accounted for by children’s externalizing problems. 

Taken together, the findings underscore that (1) maternal depressive symptoms may be associated with more hostile parenting primarily through an indirect pathway involving children’s externalizing problems; (2) maternal depressive symptoms may be directly associated with less nurturant parenting; (3) maternal depressive symptoms may be associated with increased child screen time through children’s externalizing problems; and (4) children’s internalizing problems do not appear to function as developmental mechanisms linking maternal depressive symptoms with parenting quality and/or child screen time. 

### 4.1. Children’s Externalizing Problems as a Pathway Linking Maternal Depressive Symptoms to Parenting Quality

The significant indirect pathways observed in this study were underpinned by the positive association between maternal depressive symptoms and children’s internalizing and externalizing problems (i.e., the first stage of the mediation models), consistent with the well-established association between maternal depressive symptoms and children’s behavioural outcomes [3]. Maternal depressive symptoms commonly involve fatigue, withdrawal, and unresponsiveness [73], which may interfere with mothers’ ability to response consistently and sensitively to their children [34,35]. In turn, children may begin to exhibit behaviours characteristic of internalizing and externalizing problems in attempts to self-regulate and/or evoke different responses from their mothers [41,52]. Externalizing behaviours have specifically been theorized to be potentially adaptive responses that buffer the allostatic load of adversity on physiological outcomes [74]. Similarly, internalizing behaviours may emerge as learned responses, whereby children whose mothers are experiencing depressive symptoms direct their distress inwardly in an attempt to self-regulate when caregivers’ support cannot be reliably attained [41,75,76]. The consistent positive association between mothers’ depressive symptoms and their preschool children’s behavioural problems, observed in this study and previous research, highlights the importance of addressing mothers’ depressive symptoms through primary, secondary, and tertiary preventive efforts to reduce children’s risk of developing behavioural difficulties. 

Consistent with previous research examining the second pathway of the mediation model (i.e., child behaviour to parenting quality) [7,8,9], maternal depressive symptoms were associated with more hostile parenting through greater levels of child externalizing problems. Although previous research suggests that children’s externalizing problems may elicit harsher parenting behaviours [7,8,9], to our knowledge, this is the first study to directly examine whether children’s behavioural problems mediate the association between maternal depressive symptoms and hostile parenting, providing evidence for a theoretically plausible, developmental pathway. 

Mothers experiencing depressive symptoms during pregnancy and postpartum may experience difficulties engaging with, comforting, and being present with their child, particularly when children begin to display externalizing behaviours [77,78]. This may increase children’s risk of or further reinforce the likelihood of externalizing problems as a negative bid for interaction [79], proximity-seeking behaviour [80], or self-regulation [74]. Consequently, children displaying externalizing behaviours may be perceived as more difficult to manage and to comfort emotionally, potentially resulting in more hostile parenting behaviours such as yelling, withdrawal, or physical discipline [77,78]. Although this may be the first study to examine this specific mediation pathway, the individual associations are empirically supported [3,7,8,9], and the broader developmental framework is supported by theoretical models of reciprocal parent–child influences [5,6,78]. Future research should examine whether this developmental mediation pathway is evident across diverse populations and settings, including both low- and high-risk samples within and beyond Canada.

Although no indirect effects through children’s behavioural problems were identified when examining nurturant parenting quality, significant total and direct effects indicated that maternal depressive symptoms were associated with less nurturant parenting quality. Previous research has identified significant, although generally weak associations between maternal depressive symptoms and nurturant parenting behaviours [39]. Maternal depressive symptoms may interfere with mothers’ ability to remain attuned and responsive to their young children, potentially resulting in fewer warm and supportive behaviours and thus lower quality parent–child interactions independent of children’s behavioural problems [77,78]. Thus, while maternal depressive symptoms may not necessarily contribute directly to hostile or inconsistent parenting, they may reduce mothers’ engagement in supportive and affirmative parenting behaviours, which are important for children’s development of a positive image and high self-esteem [81].

Taken together, findings from the first model have several implications for public health. Primary preventive efforts may focus on reducing the emergence of maternal depressive symptoms to decrease children’s risk of developing behavioural problems, particularly externalizing difficulties, while promoting more nurturant parenting behaviours. Secondary preventive strategies may involve screening mothers for depressive symptoms and children for externalizing problems, followed by targeted supports to reduce child-related stressors that may contribute to hostile parenting responses. Finally, a tertiary preventive strategy may include parenting interventions for mothers experiencing depressive symptoms, as evidence suggests that parenting quality can improve through intervention even when maternal depressive symptoms persist [82].

### 4.2. Children’s Externalizing Problems as a Pathway Linking Maternal Depressive Symptoms to Child Screen Time

The second model applied parallel serial mediation to examine whether maternal perinatal depressive symptoms were associated with child screen time at 5 years of age through children’s internalizing or externalizing problems, followed by independent or serial mediation via each parenting quality dimension. Although no serial mediation pathways were identified, regardless of the parenting quality dimension, an indirect effect through children’s externalizing problems was consistently observed across models. The corresponding total indirect effects were primarily attributable to the independent pathway linking maternal depressive symptoms to child screen time through children’s externalizing problems. These findings are partially consistent with the existing literature [10,83]. 

A recent meta-analysis of 117 studies demonstrated an overall association between children’s screen time (defined as exposure more broadly) and socioemotional problems; however, more detailed analyses revealed weak and non-significant (or marginally significant) associations with externalizing problems [10]. Specifically, use of screens above regional recommendations demonstrated only weak associations with children’s behavioural outcomes [10]. These weak and non-significant findings were attributed to the possibility of reverse causation [10], whereby increased children’s screen time may instead emerge as a consequence of children’s behavioural problems, either as a coping strategy or as a way that parents manage their children’s behaviour, [11,12], rather than as a cause of children’s behavioural difficulties. The findings of the present study are consistent with this interpretation and extend the literature. The novelty of this study lies in its mediation model, which includes theoretically structured and plausible variables. 

Several mechanisms may explain why children’s externalizing problems mediated the association between maternal depressive symptoms and child screen time. Mothers experiencing depressive symptoms may experience higher levels of parental stress [84] and lower perceived self-efficacy in managing their children [85], which may make it more difficult to manage children’s behaviour during the transition to bedtime without relying on screen-based activities [27,28]. Limiting children’s screen time may be particularly challenging when screen time is used to manage behavioural dysregulation and the removal of devices elicits externalizing behaviours such as tantrums or aggression [86], especially before bedtime [19,20,21,22,23]. Alternately, the observed mediation pathway may reflect children’s own use of screens as a strategy for emotional and behavioural self-regulation [87]. As noted previously, maternal depressive symptoms may reduce mothers’ engagement with previously enjoyed activities with their children and contribute to emotional withdrawal and reduced responsiveness [41]. Consequently, children may spend more time using screens to occupy themselves or self-regulate [53], while less parental monitoring or limit-setting may contribute to greater screen time for children [41,88].

The absence of independent or serial mediation through parenting quality was unexpected, particularly given evidence suggesting that parenting-related constructs may explain the associations between maternal depressive symptoms and/or child externalizing problems and child screen time [41]. Instead, these findings suggest that children’s externalizing problems may contribute more directly to increased screen time before bedtime than through changes in parenting behaviours. Specifically, the association between maternal depressive symptoms and child screen time may be mediated by children’s externalizing problems, with screen time serving as a means of self-regulation, distraction, or entertainment. However, because these mediation pathways were examined in an observational study, additional longitudinal and experimental research is needed to determine the directionality of these associations and clarify the mechanisms underlying them.

### 4.3. Limitations and Future Research Directions

This study was comprised mainly of non-racialized mothers who reported high total annual household incomes and educational attainment, all of which may have served to buffer the effects of maternal depressive symptoms on their preschool children’s behavioural problems [89,90]. Although this may limit the generalizability of this study, findings may extend to populations with similar characteristics. 

Next, our measure of screen time, which was quantified using a single-item questionnaire by mothers and was defined as the number of days a week that children used a screen for more than 1 hour, may be subject to reporting bias and not necessarily be reflective of other components of screen use (e.g., time of use, type of use). Notably, the screen time measure in the present study specifically captured screen time before bedtime and may therefore be more appropriately interpreted as an indicator of sleep hygiene practices, rather than a general measure of screen exposure. Also, screen time was measured approximately 10 years ago, which needs to be considered when contextualizing the findings due to technological advancements. Future research should investigate different components of screen use as outcomes in the investigated mediation pathways to replicate and extend our findings, considering the advancements in technology. 

The hostile parenting subscale had a higher degree of missingness than the consistency and nurturant parenting subscales. Closer inspection of the data indicated that this was largely driven by a single item, for which many mothers selected “don’t know” (i.e., “Of all the times that you talk to him/her about his/her behaviour, what proportion is disapproval?”). Although this level of missingness is not ideal, its concentration within a single item rather than across the scale suggests that respondents may have found the wording or interpretation of this item unclear, while the remaining items likely continued to capture the intended construct of hostile parenting. This is further supported by a moderate-to-high McDonald’s omega internal consistency reliability estimate. 

Lastly, the first mediation model established full temporal ordering with a clear exposure–outcome association, with maternal depressive symptoms assessed from pregnancy to 1 year postpartum, children’s behavioural problems measured at 3 years, and parenting quality measured at 5 years. In the second model, however, parenting quality and children’s screen time were both assessed at 5 years of age, meaning that temporality between these two variables cannot be established. Collecting repeated measures of parenting quality and children’s screen time across early childhood would strengthen inference regarding the proposed serial mediation pathway. Future research should also examine maternal screen time within these models, given its strong association with children’s screen time. 

## 5. Conclusions

This study examined children’s behavioural problems as potential developmental mechanisms linking maternal depressive symptoms with subsequent parenting quality and child screen time (specifically during the pre-bedtime period). Children’s externalizing problems emerged as a key developmental pathway underlying the associations between maternal depressive symptoms and both more hostile parenting quality and greater child screen time, highlighting the important role of children’s behavioural functioning in early family development. These findings suggest that children’s externalizing problems may represent a promising target for early intervention while reinforcing the importance of preventing and treating maternal depressive symptoms to support healthier parenting practices and more positive developmental outcomes for children.

## Figures and Tables

**Table 1:** Descriptions of nurturant, hostile, and consistent parenting quality.

Parenting Type	Description [42,43,44]
*Nurturant*	Characterized by warm, supportive, and affirming parental behaviours, with an emphasis on high-quality interactions and meaningful time shared between a parent and their child. It is considered a positive component of parenting.
*Hostile*	Characterized by negative emotionality directed toward the child, typically expressed through punitive, disapproving, or antagonizing behaviours. Parents exhibiting hostile parenting often display irritation, frustration or annoyance during interactions with their child.
*Consistent*	Characterized by consistency in parenting behaviours, particularly in the establishment of routine, expectations and responses to children’s requests.

**Table 2:** Sample demographic characteristics (N = 373).

Demographic Information	n (%)	Mean (SD)	Missing (n, (%))
*Total Annual Household Income ($ CAD)*			3 (0.80)
Less than 10,000	3 (0.80)	-	
20,000–39,999	7 (1.88)	-	
40,000–69,999	34 (9.12)	-	
70,000–100,000	87 (23.32)	-	
More than 100,000	239 (64.08)	-	
*Education Level*			1 (0.27)
Completed Post-Graduate Degree	104 (27.88)	-	
Completed University Degree	185 (49.60)	-	
Completed Trade/Technical School	63 (16.89)	-	
Completed High School Diploma	20 (5.36)	-	
*Self-Identified Ethnicity*			0 (0.00)
Non-Racialized	340 (91.15)	-	
Racialized	33 (8.85)		
*Children’s Sex Assigned at Birth*			0 (0.00)
Male	189 (50.67)	-	
Female	184 (49.33)	-	
*Age, Birthweight, and Gestational Age at Birth*			
Age of Mother (years)	-	37.94 (3.70)	0 (0.00)
Age of Child (years)	-	5.11 (0.19)	0 (0.00)
Children’s Birthweight (grams)	-	3368.40 (520.06)	2 (0.54)
Children’s Gestational Age at Birth (weeks)	-	39.26 (1.78)	1 (0.27)

**Table 3:** Characteristics of study variables (N = 373).

Study Variable	n (%)	Mean (SD)	Missing (n, %)
***Predictor***			
Maternal Mean Depressive Symptoms		4.57 (3.11)	1 (0.27)
***Mediators***			
Parenting Quality			
Nurturant	-	14.97 (2.49)	7 (1.88)
Hostile	-	9.40 (3.39)	160 (42.90)
Consistent	-	16.27 (2.69)	37 (9.92)
Number of Days Children Engaged in ≥1 h of Screen Time Before Bed			5 (1.34)
0	71 (19.03)	-	
1	69 (18.50)	-	
2	57 (15.28)	-	
3	25 (6.70)	-	
4	26 (6.97)	-	
5	28 (7.51)	-	
6	15 (4.02)	-	
7	77 (20.64)	-	
***Outcome***			
Children’s Behavioural Problems			2 (0.54)
Internalizing Problems (T Score)	-	45.43 (8.76)	
Externalizing Problems (T score)	-	45.48 (8.70)	

## Data Availability

The data associated with this study have not been deposited into a publicly available repository; however, the data will be made available on request to the corresponding author.

## Supplementary Materials

The following supporting information can be downloaded at: https://www.mdpi.com/article/10.3390/children13091144/s1, Figure S1: Participant flow diagram; Table S1: Parallel mediation models examining children’s behavioural problems as mediators of associations between maternal depressive symptoms and parenting quality; Table S2: Parallel serial mediation models examining pathways from maternal depressive symptoms to child screen time through children’s behavioural problems and parenting quality.
