# Supplementary material for: Developmental Pathways Linking Maternal Depressive Symptoms with Parenting Quality and Preschool Children’s Screen Time: The Role of Children’s Behavioural Problems
Source: Children (Basel). 2026 Aug 26;13(9):1144. doi: 10.3390/children13091144 (PMC13605011; doi:10.3390/children13091144)
Supplement: Supplementary file 1 [file children-13-01144-s001.zip › children-4371431-supplementary.pdf]

Supplementary Materials

Developmental Pathways Linking Maternal Depressive Symptoms with Parenting Quality and Preschool Children's Screen Time: The Role of Children's Behavioural Problems

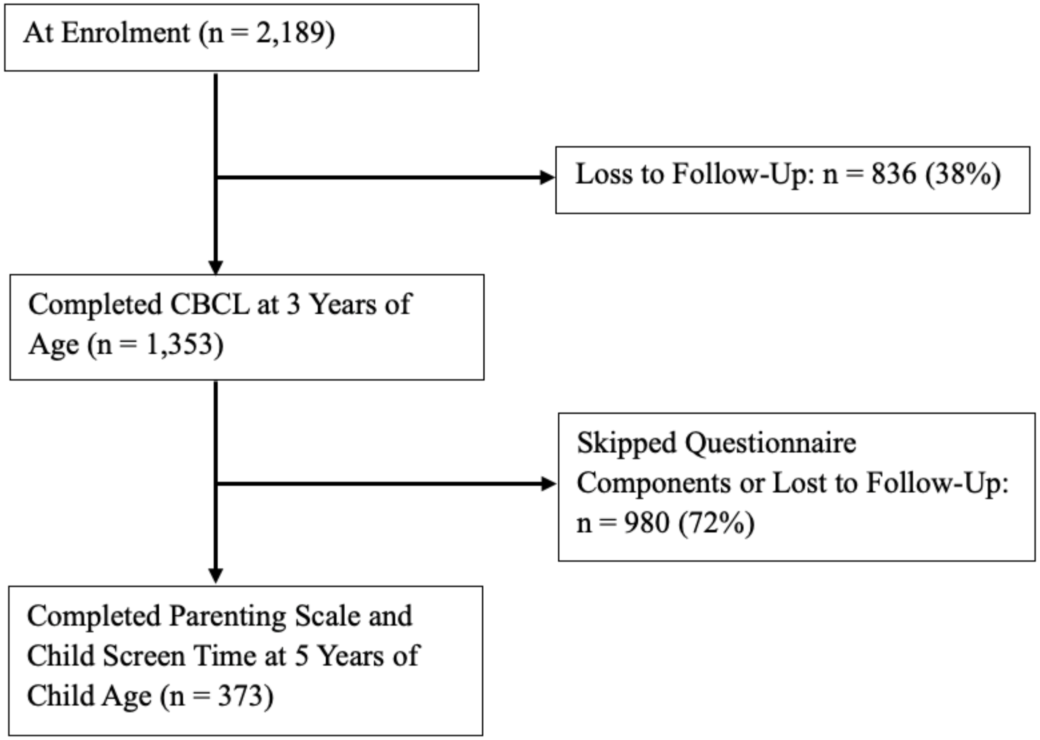

Figure S1. Participant flow diagram.

Table S1. Parallel mediation models examining children's behavioural problems as mediators of associations between maternal depressive symptoms and parenting quality.

Parenting Outcome	Model parameter/path	b	SE	t	p	95% CI	β
Nurturant Parenting	Depression → Internalizing	0.707	0.144	4.907	<0.001	[0.425, 0.990]	0.250
	Depression → Externalizing	0.487	0.145	3.359	0.001	[0.203, 0.771]	0.174
	Internalizing → Nurturant parenting	−0.001	0.019	−0.029	0.977	[−0.038, 0.037]	−0.002
	Externalizing → Nurturant parenting	−0.020	0.019	−1.040	0.298	[−0.057, 0.018]	−0.069
	Indirect via internalizing	−0.000	0.014	−0.029	0.977	[−0.027, 0.026]	−0.000
	Indirect via externalizing	−0.010	0.010	−0.981	0.327	[−0.029, 0.010]	−0.012
	Total indirect effect	−0.010	0.011	−0.883	0.377	[−0.032, 0.012]	−0.012
	Indirect-effect contrast	0.009	0.021	0.443	0.657	[−0.032, 0.050]	0.011
	Direct effect	−0.101	0.043	−2.326	0.020	[−0.186, −0.016]	−0.125
	Total effect	−0.111	0.042	−2.634	0.008	[−0.193, −0.028]	−0.138
Hostile Parenting	Depression → Internalizing	0.707	0.160	4.426	<0.001	[0.394, 1.020]	0.250
	Depression → Externalizing	0.487	0.161	3.030	0.002	[0.172, 0.802]	0.174
	Internalizing → Hostile parenting	−0.024	0.028	−0.878	0.381	[−0.079, 0.030]	−0.063
	Externalizing → Hostile parenting	0.133	0.028	4.826	<0.001	[0.078, 0.188]	0.342
	Indirect via internalizing	−0.017	0.020	−0.867	0.387	[−0.056, 0.022]	−0.016
	Indirect via externalizing	0.065	0.021	3.100	0.002	[0.024, 0.106]	0.059
	Total indirect effect	0.048	0.022	2.185	0.029	[0.005, 0.090]	0.044
	Indirect-effect contrast	−0.082	0.034	−2.382	0.018	[−0.150, −0.014]	−0.075
	Direct effect	0.041	0.063	0.660	0.510	[−0.082, 0.165]	0.038
	Total effect	0.089	0.063	1.422	0.157	[−0.034, 0.213]	0.082
Consistent Parenting	Depression → Internalizing	0.707	0.146	4.847	<0.001	[0.421, 0.993]	0.250
	Depression → Externalizing	0.487	0.147	3.318	0.001	[0.199, 0.775]	0.174
	Internalizing → Consistent parenting	0.016	0.020	0.813	0.416	[−0.023, 0.056]	0.054
	Externalizing → Consistent parenting	−0.027	0.020	−1.326	0.185	[−0.066, 0.013]	−0.086
	Indirect via internalizing	0.012	0.014	0.801	0.423	[−0.017, 0.040]	0.013
	Indirect via externalizing	−0.013	0.011	−1.196	0.232	[−0.034, 0.008]	−0.015
	Total indirect effect	−0.001	0.012	−0.113	0.910	[−0.025, 0.022]	−0.002
	Indirect-effect contrast	0.025	0.023	1.090	0.276	[−0.020, 0.069]	0.028
	Direct effect	−0.070	0.046	−1.523	0.128	[−0.159, 0.020]	−0.080
	Total effect	−0.071	0.044	−1.598	0.110	[−0.158, 0.016]	−0.082

NOTE: b = unstandardized coefficient; SE = standard error; β = standardized coefficient.

Table S2. Parallel serial mediation models examining pathways from maternal depressive symptoms to child screen time through children's behavioural problems and parenting quality.

Parenting Pathway	Model parameter/path	b	SE	t	p	95% CI	β
Nurturant Parenting	Depression → Internalizing	0.707	0.144	4.907	0.000	[0.425, 0.990]	0.250
	Depression → Externalizing	0.487	0.145	3.359	0.001	[0.203, 0.771]	0.174
	Internalizing → Nurturant parenting	−0.001	0.019	−0.029	0.977	[−0.038, 0.037]	−0.002
	Externalizing → Nurturant parenting	−0.020	0.019	−1.040	0.298	[−0.057, 0.018]	−0.069
	Depression → Nurturant parenting	−0.101	0.043	−2.326	0.020	[−0.186, −0.016]	−0.125
	Internalizing → Screen time	−0.019	0.019	−0.973	0.330	[−0.057, 0.019]	−0.064
	Externalizing → Screen time	0.068	0.019	3.551	0.000	[0.031, 0.106]	0.229
	Nurturant parenting → Screen time	−0.065	0.053	−1.226	0.220	[−0.168, 0.039]	−0.062
	Indirect via internalizing	−0.013	0.014	−0.942	0.346	[−0.041, 0.014]	−0.016
	Indirect via externalizing	0.033	0.014	2.418	0.016	[0.006, 0.060]	0.040
	Indirect via parenting	0.007	0.006	1.165	0.244	[−0.004, 0.018]	0.008
	Serial indirect via internalizing → parenting	0.000	0.001	0.054	0.957	[−0.001, 0.001]	0.000
	Serial indirect via externalizing → parenting	0.001	0.001	0.854	0.393	[−0.001, 0.002]	0.001
	Total indirect via internalizing	−0.013	0.014	−0.939	0.348	[−0.041, 0.014]	−0.016
	Total indirect via externalizing	0.034	0.014	2.445	0.014	[0.007, 0.061]	0.041
	Overall total indirect effect	0.027	0.015	1.801	0.072	[−0.002, 0.057]	0.032
	Behaviour indirect-effect contrast	−0.047	0.024	−1.928	0.054	[−0.094, 0.001]	−0.056
	Serial indirect-effect contrast	−0.001	0.001	−0.533	0.594	[−0.003, 0.002]	−0.001
	Total behaviour indirect-effect contrast	−0.047	0.024	−1.945	0.052	[−0.095, 0.000]	−0.056
	Direct effect	0.054	0.044	1.230	0.219	[−0.032, 0.141]	0.065
	Total effect	0.081	0.043	1.881	0.060	[−0.003, 0.166]	0.097
Hostile Parenting	Depression → Internalizing	0.707	0.156	4.529	0.000	[0.401, 1.013]	0.250
	Depression → Externalizing	0.487	0.157	3.100	0.002	[0.179, 0.795]	0.174
	Internalizing → Hostile parenting	−0.024	0.027	−0.899	0.370	[−0.078, 0.029]	−0.063
	Externalizing → Hostile parenting	0.133	0.027	4.938	0.000	[0.079, 0.187]	0.342
	Depression → Hostile parenting	0.041	0.061	0.676	0.500	[−0.080, 0.163]	0.038
	Internalizing → Screen time	−0.018	0.021	−0.845	0.398	[−0.059, 0.023]	−0.060
	Externalizing → Screen time	0.065	0.022	2.986	0.003	[0.022, 0.107]	0.217
	Hostile parenting → Screen time	0.036	0.044	0.815	0.417	[−0.051, 0.122]	0.047
	Indirect via internalizing	−0.013	0.015	−0.819	0.413	[−0.043, 0.017]	−0.015
	Indirect via externalizing	0.032	0.015	2.127	0.033	[0.002, 0.061]	0.038
	Indirect via parenting	0.002	0.004	0.428	0.670	[−0.006, 0.009]	0.002
	Serial indirect via internalizing → parenting	−0.001	0.001	−0.808	0.421	[−0.003, 0.001]	−0.001
	Serial indirect via externalizing → parenting	0.002	0.002	1.042	0.299	[−0.002, 0.007]	0.003
	Total indirect via internalizing	−0.013	0.015	−0.865	0.387	[−0.043, 0.017]	−0.016
	Total indirect via externalizing	0.034	0.015	2.252	0.024	[0.004, 0.063]	0.040
	Overall total indirect effect	0.022	0.016	1.431	0.153	[−0.008, 0.053]	0.026
	Behaviour indirect-effect contrast	−0.044	0.026	−1.683	0.092	[−0.095, 0.007]	−0.053
	Serial indirect-effect contrast	−0.003	0.003	−1.099	0.274	[−0.009, 0.003]	−0.004
	Total behaviour indirect-effect contrast	−0.047	0.026	−1.792	0.073	[−0.099, 0.004]	−0.056

**Parenting Pathway**	**Model parameter/path**	**b**	**SE**	**t**	**p**	**95% CI**	**β**
	Direct effect	0.059	0.048	1.245	0.213	[−0.034, 0.152]	0.071
	Total effect	0.081	0.047	1.733	0.083	[−0.011, 0.174]	0.097
Consistent Parenting	Depression → Internalizing	0.707	0.146	4.854	0.000	[0.422, 0.993]	0.250
	Depression → Externalizing	0.487	0.147	3.323	0.001	[0.200, 0.774]	0.174
	Internalizing → Consistent parenting	0.016	0.020	0.814	0.416	[−0.023, 0.056]	0.054
	Externalizing → Consistent parenting	−0.027	0.020	−1.328	0.184	[−0.066, 0.013]	−0.086
	Depression → Consistent parenting	−0.070	0.046	−1.525	0.127	[−0.159, 0.020]	−0.080
	Internalizing → Screen time	−0.017	0.019	−0.859	0.390	[−0.055, 0.021]	−0.056
	Externalizing → Screen time	0.066	0.019	3.422	0.001	[0.028, 0.104]	0.222
	Consistent parenting → Screen time	−0.126	0.051	−2.466	0.014	[−0.226, −0.026]	−0.130
	Indirect via internalizing	−0.012	0.014	−0.835	0.404	[−0.040, 0.016]	−0.014
	Indirect via externalizing	0.032	0.014	2.389	0.017	[0.006, 0.059]	0.039
	Indirect via parenting	0.009	0.007	1.191	0.234	[−0.006, 0.023]	0.010
	Serial indirect via internalizing → parenting	−0.001	0.002	−0.675	0.500	[−0.006, 0.003]	−0.002
	Serial indirect via externalizing → parenting	0.002	0.002	0.935	0.350	[−0.002, 0.005]	0.002
	Total indirect via internalizing	−0.013	0.014	−0.925	0.355	[−0.041, 0.015]	−0.016
	Total indirect via externalizing	0.034	0.014	2.414	0.016	[0.006, 0.062]	0.041
	Overall total indirect effect	0.029	0.016	1.854	0.064	[−0.002, 0.060]	0.035
	Behaviour indirect-effect contrast	−0.044	0.024	−1.837	0.066	[−0.091, 0.003]	−0.053
	Serial indirect-effect contrast	−0.003	0.004	−0.886	0.376	[−0.010, 0.004]	−0.004
	Total behaviour indirect-effect contrast	−0.047	0.025	−1.919	0.055	[−0.095, 0.001]	−0.056
	Direct effect	0.052	0.044	1.181	0.238	[−0.034, 0.139]	0.062
	Total effect	0.081	0.044	1.853	0.064	[−0.005, 0.168]	0.097

Note. b = unstandardized coefficient; SE = standard error; β = standardized coefficient.
